# Ethnic disparities in tuberculosis incidence and related factors among indigenous and other communities in ethnically diverse Suriname

**DOI:** 10.1016/j.jctube.2021.100227

**Published:** 2021-03-10

**Authors:** F.A. Gopie, A. Hassankhan, S. Ottevanger, I. Krishnadath, W. de Lange, C.W.R. Zijlmans, S. Vreden

**Affiliations:** a Pulmonologist, Academic Hospital Paramaribo, Paramaribo, Suriname; b Amsterdam UMC, University of Amsterdam, Medical Microbiology, Amsterdam, The Netherlands; c Anton de Kom University of Suriname, Faculty of Medical Sciences, Paramaribo, Suriname; d University of Groningen, University Medical Center Groningen, Department of Pulmonary Diseases and Tuberculosis, Groningen, The Netherlands; e University of Groningen, University Medical Center Groningen, Tuberculosis Center Beatrixoord, Haren, The Netherlands; f Department of Paediatrics, Diakonessenhuis Hospital, Paramaribo, Suriname; g Department of Internal Medicine, Academic Hospital Paramaribo, Paramaribo, Suriname; h MoleMann Mental Health Suriname, Paramaribo, Suriname

**Keywords:** Ethnicity, Indigenous, HIV, Poverty

## Abstract

**Background:**

In Suriname, a country home to many ethnic groups, a high incidence of tuberculosis (TB) has been found among Indigenous Trio Amerindians. However, whether wider ethnic disparities in TB incidence and its associated risk factors (e.g., diabetes mellitus and HIV) exist in Suriname, is not known. We sought to investigate disparities in TB incidence and its risk factors on ethnicity in Suriname, as this could give way to targeted TB intervention programs.

**Methods:**

Anonymized patient data from 2011 to 2015 was extracted from the National TB Registry and analyzed. Differences in the five-year incidence rates of TB for the six largest ethnic groups—Creole, Hindustani, Indigenous, Javanese, Maroon, and Mixed—were assessed using a chi-square goodness-of-fit test, and TB patient differences regarding ethnicity were evaluated for selected factors using a multinomial logistic regression with Creole patients as reference.

**Results:**

662 Patients were eligible for analyses with the following ethnic makeup: Creole (36.4%), Hindustani (15.6%), Indigenous (8.6%), Javanese (10.6%), Maroon (15.1%), and Mixed ethnicity (13.7%). Differences in five-year incidence rates for TB were significant, *χ*^2^(5, N = 662) = 244.42, *p* < .001, and the highest TB rates were found for Indigenous (280 per 100,000) and Creole people (271 per 100,000). HIV coinfection was a TB risk factor for Creoles (38.2% of these patients were HIV positive). Several variables (i.e., those for drug use) had high levels of incomplete or missing data.

**Conclusions:**

Our study has demonstrated that ethnic disparities in tuberculosis incidence exist in Suriname and that they are associated with specific, known risk factors such as HIV (especially for Creole people). For Indigenous people, risk factors may include diminished access to health care facilities and low socioeconomic status. However, direct data on these factors was unavailable. These findings call for targeted national intervention programs—with special attention given to the vulnerabilities of susceptible ethnic groups—and improved data collection.

## 1 Introduction

Tuberculosis (TB) is the leading cause of death from a single infectious agent. In 2018, some 10 million people fell ill with active TB and an estimated 1.5 million TB-related deaths occurred. The burden of TB, however, is an unequal one. TB is associated with poverty, overcrowding, and malnutrition [1]. This disease may also vary by ethnicity. For example, globally, Indigenous peoples are generally burdened with TB at a disproportionate rate [2]. This inequality has implications for Suriname, a highly diverse country.

Suriname is a lower middle-income country bordering northern Brazil that had a population of 541,638 in 2012 [3] and a TB incidence rate of about 38 cases per 100,000 in 2018 [4]. All cases of TB in Suriname should be reported to the National Tuberculosis Program (NTP), a government workforce that is responsible for registering TB patients and their treatment outcome nationwide, the availability of the tuberculin skin test, the free provision of TB medication through the Directly Observed Therapy program, and TB contact tracing. Screening prisoners for TB is also part of the NTP’s activities. In 2011, the NTP released the first Surinamese TB guidelines, which were recently updated according to WHO recommendations. Persons suspected of having TB are evaluated, classified, and treated with first line tuberculostatic drugs according to WHO guidelines [5].

Suriname’s multicultural society harbors numerous ethnic groups, in part due to its colonial past [6]; the six largest groups are: a) Creoles (including Afro-Surinamers; 16.4%), descendants of African slaves that may also have European and other ancestors; b) Hindustanis (27.4%), descendants of contract laborers from what was then British India; c) Indigenous Amerindians (3.7%), a group comprising multiple tribes (e.g., the Trio, Wayana, and Akurio); d) Javanese (13.7%), descendants of contract laborers predominantly from Java, Indonesia (then the Dutch East Indies); e) Maroons (21.7%), descendants of escaped African slaves that mostly settled Suriname’s interior; and f) individuals of Mixed ethnicity (13.4%). Many Indigenous and Maroon people continue to live in tribal communities spanning Suriname’s remote, densely forested interior and continue to maintain their cultural beliefs and traditions. Almost 90% of Suriname’s inhabitants live in the more accessible coastal area. The remaining population lives in the interior, which is mainly accessible by air and river transportation. Suriname’s capital, Paramaribo, is also located near the coast and is home to 45% of the population [3], [7].

Regarding TB, the Indigenous population in Suriname deserves special consideration. In neighboring Brazil, higher rates of TB among Indigenous peoples were associated with poverty, limited living space, and limited healthcare access [8]. Globally, Indigenous Amazonian groups are among the worst affected populations [2].

Suriname is home to Indigenous communities that share similarities with their Brazilian counterparts and therefore compose a potentially vulnerable group regarding TB. An investigation by van Crevel *et al.* assessing TB cases between 1995 and 2000 among Trio Indians living in Kwamalasamutu—a village remotely situated in Suriname’s tropical rainforest interior near the southern border with Brazil—revealed a higher incidence and familial clustering of TB. These observations were attributed to lifestyle, a possible genetic predisposition for TB, and limited access to healthcare among the Trio Indians [9], who live in relative isolation [10]. Tollefson *et al.* estimated that the prevalence of TB among this community was seven times higher than Suriname’s national comparison rate [2].

In the past two decades TB rates have been on the rise in Suriname [11]. This increase has probably been fueled by HIV, with most HIV positive patients being of Creole and Maroon descent [12]. This led to collaborative programs to address the dual health threat. People living with HIV (PLHIV) and presenting with prolonged cough and or weight loss are referred to the NTP for free of charge tuberculin skin testing and sputum examination. Considering this observation, the high incidence of TB in Suriname’s Indigenous communities, and the significantly higher prevalence of diabetes mellitus (DM; a TB risk factor) among Hindustanis [13], we investigated differences between ethnic groups on TB incidence and risk factors in Suriname, as this could contribute to a better understanding of the state of TB in Suriname and give way to targeted TB intervention programs.

## 2 Methods

We conducted a retrospective cohort study of National TB Registry records from January 1st, 2011 to December 31st, 2015. To that end, relevant information on ethnicity, age, sex, HIV status, DM status, substance abuse, alcohol consumption, smoking, TB presentation (pulmonary or extra pulmonary TB), case type, and outcome was extracted from the NTP register.

Genotyping of TB strains is not available in Suriname. As a result, it is not possible to determine whether patients referred for retreatment of TB have been infected with another TB strain or if their previous TB infection has flared up. Therefore, only the first entry of a patient in the NTP register during the study period was considered for analysis. Consequently, of patients with multiple entries in the NTP registry during the assessed timeframe (due to, for example, relapse or resumption of treatment after loss to follow-up), only the first entry was included in analyses.

### 2.1 Ethics

This study was conducted with the approval of the Ministry of Health (letter #VG 010-15). Only retrospective data was considered, and the dataset used for analyses was anonymized.

### 2.2 Tuberculosis testing algorithm and patient work-up

Patients suspected of having TB (i.e., presenting with prolonged cough, fever, night sweats, and weight loss) can have their sputum collected locally at their residence. The sputum is thereafter sent to Paramaribo for further diagnostic evaluation, be it at the Academic Hospital or the Central Laboratory of the Bureau of Public Health. If acid-fast bacilli are detected in the sputum or clinical suspicion of TB arises, patients are referred by their physician or local health official (or nursing aide in Suriname’s interior) to the capital, Paramaribo, for further evaluation and treatment by a pulmonologist, infectious disease specialist or pediatrician.

Most patients are referred to pulmonologists, who practice in the sole national pulmonary medicine clinic at the Academic Hospital in Paramaribo. Evaluation of patients suspected of TB includes a detailed anamnesis and chest X-ray examination, with emphasis on upper lobe consolidation, miliary consolidation, cavitary lesions, confluencing patchy infiltrates, enlarged mediastinal lymph nodes, and/or pleural effusion. Additional diagnostic testing consists of sputum examinations for acid-fast bacilli (AFB) in Ziehl-Neelsen or Auramine staining and, from May 2012 onwards, Xpert MTB/RIF (Cepheid, Sunnyvale, CA, USA) tests on a patient’s first sputum sample. In young children unable to cough on demand, empty stomach aspirate is obtained thrice and examined. Occasionally a tuberculin skin test is performed, with a result of 10 mm or more considered positive for those in the general population and 5 mm or more considered positive for PLHIV or children under 5 years of age. Tuberculous meningitis is assumed on clinical presentation and the ruling out of other central nervous system infections based on the chemical, cellular, and bacteriological composition of cerebrospinal fluid [5]. Patients are assigned a diagnosis of pulmonary TB mainly based on the sputum test results or on clinical presentation.

Children up to 15 years of age are treated by pediatricians and most adult patients are admitted by pulmonologists (at the sanatorium of the Academic Hospital Paramaribo) or occasionally treated by an internal medicine specialist. After discharge patients are given an outpatient appointment with their medical specialist, and daily visits by DOT-supporters are conducted (e.g., to maintain medication supplies and adherence).

### 2.3 Study variables

The study variables were extracted from the NTP records, the main variable being self-reported ethnicity. The categories of ethnicity that were assessed—regarding individuals of Creole, Hindustani, Indigenous, Javanese, Maroon, and Mixed ethnicity—all corresponded to ethnic or racial categories used in the 2012 census [7], [14]. Due to the paucity of patients from other ethnic or racial groups, individuals not belonging to one of these six categories were omitted from analysis.

Other variables investigated as factors were: age (measured in years), sex (female, male), HIV status (negative, positive, unknown), DM status (no, yes; fasting blood glucose [FBG] level ≥ 6.5 mmol/l—when TB patients with DM were admitted, their FBG level was measured twice a week), substance abuse (no, yes), alcohol consumption (no, yes), and smoking (no, yes).

### 2.4 Statistics

Information—on age, sex, HIV status, DM status, substance abuse, alcohol consumption, and smoking—was compiled and tabulated for each of the six assessed ethnic groups. Except for age, which was used as a continuous variable, all other variables were categorical. For analyses that included HIV status as a variable, patients with an unknown HIV status were excluded (listwise deletion). TB manifestation was excluded from analysis due to the difficulty in correctly diagnosing extrapulmonary TB in Suriname [15].

Independent univariate analyses were performed to gauge the association between each of the remaining variables and ethnicity. For age, a continuous variable, this was a one-way ANOVA. For the categorical variables, sex and HIV status, chi-square tests were used.

For each ethnic group, the five-year incidence rate for TB was calculated by dividing the number of unique patients of an assessed ethnicity entered in the NTP registry during the study period by the number of individuals of that ethnicity living in Suriname according to the 2012 census. These incidence rates, based on the patient population’s ethnic makeup, were then compared to the expected five-year incidence rates based on the demographic breakdown of the 2012 census using a chi-square goodness-of-fit test. Incidence rates were reported per 100,000 population. A similar analysis, limited to HIV negative patients, was also performed.

A multinomial logistic regression was used to evaluate selected factors against the six assessed ethnic groups. Age, sex, and HIV status were used as predictors. Cases involving Creole patients were used as a benchmark because, compared to the other ethnic groups, they contributed the largest number of eligible TB patients (n = 241).

Statistical analyses were performed using SPSS version 20.0 (computer software; IBM Corp., 2011). For significance testing, all alpha levels were set at 0.05.

## 3 Results

Between 2011 and 2015, a combined total of 716 new and retreatment TB cases were reported to the NTP. Table 1 shows the number of TB cases by ethnicity per year. At presentation, a diagnosis of TB was assumed in 61% of cases based on the presence of acid-fast bacilli. In the remaining 39%, a TB diagnosis was assumed on basis of clinical presentation. From May 2012 onwards, an average of 64% of all TB diagnoses were confirmed through GeneXpert tests—approximately 72% of pulmonary TB and 12% of extrapulmonary TB cases were confirmed by PCR test. Culture was done in 502 cases with growth of bacilli detected in 368 cases (73%). We performed our analysis on unique patients in the NTP database. Consequently, one case with incomplete data was excluded, leaving 715 cases (adult, n = 683; pediatric, n = 32). Further excluded from analysis were (a) 15 cases concerning patients of Chinese, European, or Brazilian descent (due to the small number of cases concerning these groups) and (b) 38 subsequent repeat cases of patients that were recorded twice or thrice in the NTP register during the study period (due to their resumption of treatment after being lost to follow-up). Analyses were performed using the remaining 662 cases, which corresponded to 662 unique patients (Table 2), of whom 569 were classified as pulmonary TB and 93 as extra pulmonary TB.

**Table 1:** TB cases by ethnicity between 2011 and 2015.

Group/Year	2011	2012	2013	2014	2015
Creole	59	52	45	60	46
Hindustani	17	30	16	20	22
Maroon	19	17	22	27	20
Mixed	14	9	28	24	20
Javanese	9	15	18	12	21
Indigenous	11	11	10	12	14
Chinese	0	0	2	2	6
European	2	1	0	1	0
Brazilian	1	0	0	0	0
No data	0	0	0	0	1
Total	132	135	141	158	150

**Table 2:** Breakdown by ethnicity of variables recorded for TB patients.

Variable^1^	Category	Creole		Hindustani		Indigenous		Javanese		Maroon		Mixed		Total	
Age^†^, *p* < .001	Years (SD)	44	(13.6)	44.1	(15.5)	38	(18.5)	49.9	(16.2)	33.8	(16.5)	37.5	(17.6)	41.7	(16.3)
Sex	Male	175	(72.6)	86	(83.5)	37	(64.9)	49	(70)	65	(65)	58	(63.7)	470	(71)
*p* = .021^‡^	Female	66	(27.4)	17	(16.5)	20	(35.1)	21	(30)	35	(35)	33	(36.3)	192	(29)
HIV	Negative	142	(58.9)	73	(70.9)	49	(86)	68	(97.1)	73	(73)	60	(65.9)	465	(70.2)
*p* < .001^‡^	Positive	92	(38.2)	24	(23.3)	7	(12.3)	1	(1.4)	24	(24)	27	(29.7)	175	(26.4)
	No data*	7	(2.9)	6	(5.8)	1	(1.8)	1	(1.4)	3	(3)	4	(4.4)	22	(3.3)
Diabetes	Positive	11	(4.6)	13	(12.6)	12	(21.1)	19	(27.1)	5	(5)	7	(7.7)	67	(10.1)
	No data	230	(95.4)	90	(87.4)	45	(78.9)	51	(72.9)	95	(95)	84	(92.3)	595	(89.9)
Substance abuse	Yes	77	(32)	29	(28.2)	12	(21.1)	14	(20)	32	(32)	27	(29.7)	191	(28.9)
	No data	164	(68)	74	(71.8)	45	(78.9)	56	(80)	68	(68)	64	(70.3)	471	(71.1)
Alcohol consumption	Yes	46	(19.1)	24	(23.3)	19	(33.3)	9	(12.9)	24	(24)	27	(29.7)	149	(22.5)
	No data	195	(80.9)	79	(76.7)	38	(66.7)	61	(87.1)	76	(76)	64	(70.3)	513	(77.5)
Smoking	Yes	59	(24.5)	25	(24.3)	14	(24.6)	15	(21.4)	19	(19)	27	(29.7)	159	(24)
	No data	182	(75.5)	78	(75.7)	43	(75.4)	55	(78.6)	81	(81)	64	(70.3)	503	(76)

Notes. Values within brackets give the percentage breakdown of categories within a variable for an ethnic group.

† One-way ANOVA, *p*-value

‡ Chi-square test, *p*-value

* Category excluded from Chi-square analysis

All patients belonged to one of the six assessed ethnic groups: a) 241 (36.4%) were Creole; b) 103 (15.6%) were Hindustani; c) 57 (8.6%) were Indigenous; d) 70 (10.6%) were Javanese; e) 100 (15.1%) were Maroon; and f) 91 (13.7%) were of Mixed ethnicity. 175 patients (26.4%) were HIV positive*,* of whom 138 had a known CD4 count, with the average count being 174/mm^3^ and most patients (92.8%) having a count below the 500/mm^3^ threshold. Also, of the 175 HIV/TB patients, 109 were on ART—86 of these patients had a known CD4 count, with the average count being 154/mm^3^ and almost all patients (97.7%) having a count below the 500/mm^3^ threshold. NTP records also show that: a) 67 patients (10.1%) had a positive DM status; b) 191 (28.9%) used drugs (i.e., substance abuse); c) 149 (22.5%) consumed alcohol; and d) 159 (24%) smoked cigarettes. Because data on these last four variables were often missing or inconclusive, we omitted them from quantitative analysis (Table 2).

The number of TB patients per ethnic group and the five-year incidence for TB by ethnicity are shown in Table 3. The lowest incidence rate was observed among Hindustani people (69 per 100,000). The highest incidence rates were found among Indigenous (280 per 100,000) and Creole people (271 per 100,000). A chi-square goodness-of-fit test was used to compare the patient population’s ethnic makeup to that of Suriname’s population. The observed distribution of TB patients did not match the expected distribution, *χ*^2^(5, N = 662) = 244.42, *p* < .001 (Table 3). Regarding the HIV negative cohort, the five-year incidence for TB was also highest in Indigenous and Creole people and lowest in Hindustani people (Table 4). Post hoc testing revealed that Creole and Indigenous patients had a significantly higher TB incidence than all other groups in both the general (Table 3) and HIV negative cohort (Table 4). No broad differences were apparent between HIV positive and HIV negative patients regarding age and sex (Table 5).

**Table 3:** Nationwide five-year incidence of TB by ethnic group from 2011 through 2015.

Ethnic group^1234^	Pop. Reference, # (%)^123^	TB cases, # (%)	TB cases, 95% CI (low, high)	5-year TB inc.^5^, 95% CI (low, high)
Creole^ABCD^	88,856 (17)	241 (36.4)	(31.5, 41.3)	271 (235, 308)
Hindustani^AFF^	148,433 (28.5)	103 (15.6)	(11.9, 19.3)	69 (53, 86)
Indigenous^EGHI^	20,344 (3.9)	57 (8.6)	(5.7, 11.5)	280 (187, 374)
Javanese^BG^	73,975 (14.2)	70 (10.6)	(7.4, 13.7)	95 (66, 123)
Maroon^CH^	117,567 (22.5)	100 (15.1)	(11.4, 18.8)	85 (64, 106)
Mixed^DFI^	72,340 (13.9)	91 (13.7)	(10.2, 17.3)	126 (94, 158)
Total	521,515 (100)	662 (100)	---	127

Notes.

^1^A chi-square goodness-of-fit test was used to compare the patient population’s ethnic composition to that of Suriname’s total population—

*χ*^2^ (5, N = 662) = 244.42, *p* < .001.

^2^Data applies to the population of Suriname and figures are based on the 2012 census. According to this census, Suriname had a total population of 541,638 (this figure includes 20,123 individuals belonging to groups that were not included in this analysis).

^3^Total number of individuals of a given ethnic group according to the 2012 census. In this analysis the Creole ethnic group (N = 84,933, 15.7%) and Afro-Surinamese ethnic group (N = 3923, 0.7%) have been combined.

^4^A total of 15 post hoc pairwise comparisons were performed between ethnic groups. Bonferroni corrections were applied. Pairs of uppercase letters behind the group names in superscript represent group comparisons that revealed statistically significant differences.

^5^Per 100,000 population.

TB = Tuberculosis

Pop. = Population

inc. = incidence

**Table 4:** Approximate nationwide five-year incidence of TB for HIV negative persons by ethnic group from 2011 through 2015.

Ethnic group^1234^	Pop. Reference, # (%)^123^	TB cases, # (%)	TB cases, 95% CI (low, high)	5-year TB inc.^5^, 95% CI (low, high)
Creole^ABCD^	~88100 (17)	142 (30.5)	(24.9, 36.2)	161 (132, 191)
Hindustani^AEFG^	~147100 (28.5)	73 (15.7)	(11.3, 20.1)	50 (36, 64)
Indigenous^EHIJ^	~20200 (3.9)	49 (10.5)	(6.8, 14.3)	243 (156, 329)
Javanese^BFH^	~73300 (14.2)	68 (14.6)	(10.3, 18.9)	93 (65, 120)
Maroon^CI^	~116500 (22.5)	73 (15.7)	(11.3, 20.1)	63 (45, 80)
Mixed^DGJ^	~71700 (13.9)	60 (12.9)	(8.8, 17)	84 (57, 110)
Total	~516900 (100)	465 (100)	---	90

Notes.

^1^A chi-square goodness-of-fit test was used to compare the patient population’s ethnic composition to that of Suriname’s estimated HIV negative population—

*χ*^2^ (5, N = 465) = 138.89, *p* < .001.

^2^Data applies to population of Suriname and figures are based on the 2012 census. According to this census, Suriname had a total population of 541,638 (this figure includes 20,123 individuals belonging to groups that were not included in this analysis). This analysis is limited to the population of Suriname living without HIV. Population figures were estimated by subtracting the number of PLHIV per ethnic group. This is based on an HIV figure of 0.9% [AA 11Y] (this method does not, however, account for differences in HIV prevalence between ethnic groups).

^3^Total number of individuals of a given ethnic group according to the 2012 census. In this analysis the Creole ethnic group (N = 84,933, 15.7%) and Afro-Surinamese ethnic group (N = 3923, 0.7%) have been combined.

^4^A total of 15 post hoc pairwise comparisons were performed between ethnic groups. Bonferroni corrections were applied. Pairs of uppercase letters behind the group names in superscript represent group comparisons that revealed statistically significant differences.

^5^Per 100,000 population.

TB = Tuberculosis

Pop. = Population

inc. = incidence

**Table 5:** Breakdown by ethnicity of variables recorded for TB patients by HIV status.

Variable^1^	HIV status	Category	Creole		Hindustani		Indigenous		Javanese		Maroon		Mixed		Total	
Age	Negative	Years (SD)	44	(14.9)	45.1	(16.7)	37.2	(19)	50.3	(16.5)	33	(17.1)	35.5	(18.2)	41.6	(17.6)
	Positive	Years (SD)	45	(10.2)	41	(10)	46.4	(14.8)	---	---	39.1	(9.4)	43.7	(11.5)	43.6	(10.6)
Sex	Negative	Male	107	(75.4)	57	(78.1)	33	(67.3)	47	(69.1)	46	(63)	39	(65)	329	(70.8)
		Female	35	(24.6)	16	(21.9)	16	(32.7)	21	(30.9)	27	(37)	21	(35)	136	(29.2)
	Positive	Male	62	(67.4)	23	(95.8)	3	(42.9)	1	(100)	17	(70.8)	17	(63)	123	(70.3)
		Female	30	(32.6)	1	(4.2)	4	(57.1)	0	(0)	7	(29.2)	10	(37)	52	(29.7)

Notes. Values within brackets give the percentage breakdown of categories within a variable for an ethnic group.

^1^Age information suppressed for small groups.

Regarding univariate analyses performed to explore the respective relationships between ethnicity and age, sex, and HIV status, several significant results were found (Table 2). To determine a statistically significant difference between ethnic groups on age, a one-way ANOVA was conducted. There was a significant effect of ethnicity on age for the six groups *F*(5, 656) = 12.35, *p* < .001. Chi-square tests of independence were performed to examine the relation between ethnicity and sex and HIV status, respectively. Significant relationships were found between ethnicity and sex (*χ*^2^ [5, N = 662] = 13.23, *p* = .021) and ethnicity and HIV status (*χ*^2^ [5, N = 640] = 47.64, *p* < .001; unknown cases [n = 22] excluded). In comparing ethnic groups, a multinomial logistic regression was performed to model the relationship between selected variables and ethnicity. Age, sex, and HIV status were used as predictors in this analysis. Addition of the predictors to a model containing only the intercept significantly improved model fit, *χ*^2^ (15, N = 640) = 124.3, *p* < .001, with the proportion of variance explained being: R^2^ = 0.18 (Cox & Snell)/R^2^ = 0.18 (Nagelkerke). Additionally, predictors that were found to be significant using likelihood ratio tests were age (*p* < .001) and HIV status (*p* < .001).

Multiple significant parameter estimates were found (Table 6). Regarding age, Indigenous patients were significantly younger than Creole patients (*p* = .03, aOR = 0.979, 95% CI 0.96 – 0.98), as were Maroon (*p* < .001, aOR = 0.959, 95% CI 0.943 – 0.976) and Mixed patients (*p* = .002, aOR = 0.974, 95% CI 0.957 – 0.991). Conversely, Javanese patients were significantly older than Creole patients (*p* < .008, aOR = 1.024, 95% CI 1.006 – 1.042).

**Table 6:** Evaluation of differences between TB patients grouped by ethnicity compared to creole TB patients using multinomial logistic regression.

Variable: category (reference)^†‡^	B	SE	Sig.	aOR	95% CI	
**Hindustani**						
Intercept	−0.968	0.43	0.024*			
Age, continuous	−0.004	0.008	0.646	0.996	0.98	1.012
Sex: female (male)	0.599	0.31	0.053	1.82	0.991	3.341
HIV: pos. (neg.)	−0.664	0.272	0.015*	0.515	0.302	0.877

**Indigenous**						
Intercept	−0.014	0.438	0.975			
Age, continuous	−0.021	0.01	0.03*	0.979	0.96	0.998
Sex: female (male)	−0.247	0.329	0.453	0.781	0.41	1.489
HIV: pos. (neg.)	−1.46	0.428	0.001*	0.232	0.1	0.537

**Javanese**						
Intercept	−1.619	0.475	0.001*			
Age, continuous	0.023	0.009	0.008*	1.024	1.006	1.042
Sex: female (male)	−0.317	0.319	0.321	0.729	0.39	1.362
HIV: pos. (neg.)	−3.765	1.017	0.001*	0.023	0.003	0.17

**Maroon**						
Intercept	0.987	0.365	0.007*			
Age, continuous	−0.042	0.009	0.001*	0.959	0.943	0.976
Sex: female (male)	−0.069	0.272	0.799	0.933	0.548	1.59
HIV: pos. (neg.)	−0.505	0.279	0.07	0.603	0.349	1.043

**Mixed**						
Intercept	0.357	0.388	0.357			
Age, continuous	−0.027	0.009	0.002*	0.974	0.957	0.991
Sex: female (male)	−0.203	0.276	0.461	0.816	0.475	1.401
HIV: pos. (neg.)	−0.287	0.272	0.292	0.751	0.44	1.28

Notes.

^1^Patients (n = 22) of whom the HIV status was not known were not included in this analysis.

Sig. = significance

*significant at *p* < .05

aOR = adjusted odds ratio

HIV was also a significant predictor. Compared to Creole patients, Hindustani patients were less likely to be HIV positive (*p* = .015, aOR = 0.515, 95% CI 0.302 – 0.877), as were Indigenous (*p* = .001, aOR = 0.232, 95% CI 0.1 – 0.537) and Javanese patients (*p* < .001, aOR = 0.023, 95% CI 0.003 – 0.17).

## 4 Discussion

The distribution of five-year incidence rates for TB (2011–2015) by ethnicity differed significantly from the demographic breakdown of ethnicity in Suriname’s population. The highest incidence rates were found among Indigenous and Creole people; both groups were overrepresented among TB patients. Indigenous and Creole people respectively made up 3.9% and 17% of the assessed population of Suriname but made up 8.6% and 36.4% of the assessed TB patients (Table 3). When only HIV negative patients were considered, Indigenous and Creole people still had the highest TB incidence rates. When adjusted for HIV coinfection, the very high TB incidence in Indigenous people, compared to other ethnicities becomes even more apparent, demonstrating that other risk factors are very likely implied; Table 4). Indigenous people, exhibit the highest level of material poverty in Suriname [16]. As such poor socioeconomic status, which is related to increased TB levels [17], [18], [19], possibly constitutes a risk factor for this group regarding TB. Another factor to be considered is that many Indigenous people live in remote locations with fewer medical resources. Only 20.1% of Indigenous people live in Paramaribo, while the remainder inhabit rural districts and Suriname’s interior [20]. Medical Mission, a non-governmental organization, provides much of the primary healthcare in the interior but secondary care is mostly concentrated in Paramaribo [10], [21].

Creoles had the second highest TB incidence and have the highest HIV prevalence in Suriname [22]. HIV is a known risk factor for the progression of latent TB infection to active TB disease [23], [24]. This may contribute to the elevated TB incidence rate among Creole people. The demographic makeup of Paramaribo, Suriname’s capital city, may also play a role. Increased community spreading of TB in Paramaribo is a possibility as most TB patients reside there [25]. Paramaribo is also the most densely populated Surinamese district by far [3], and increased population density may contribute to higher TB levels [18], [26]. Therefore, because most Creoles (72.4%) reside in Paramaribo [20], this group may have a higher exposure to TB.

Hindustanis had the lowest incidence of TB during our study period. This observation may be explained by this group being less exposed to TB due to their tendency to reside in more rural areas. In fact, only 37.2% of the Hindustani population lives in Paramaribo [20], where most TB transmission occurs [25]. Javanese patients had a low rate of HIV coinfection. This group is also likely to reside outside the capital; only 32% of the Javanese population lives in Paramaribo [20]. Moreover, those Hindustani and Javanese people that do live in Paramaribo are underrepresented in resorts/jurisdictions [20] that are more characterized by poor living conditions [27], a known TB risk factor [18], [19].

Indigenous, Maroon, and patients of Mixed ethnicity presented with TB at a younger age compared to Creole patients. In the general population there is an age gap between Maroons and Creoles with the former being younger on average than the latter and historically possessing a higher birthrate [3], [28]. A difference in age is relevant and may explain the lower TB rates among Maroons compared to Creoles due to reactivation of latent TB infections in older people [29]. However, material poverty among Maroons is high [16]. Also, while Maroon people are less likely to reside in Paramaribo (32.7%) than Creoles (72.4%) and even Hindustanis (37.2%) [20], those that do live in Paramaribo mostly inhabit resorts/jurisdictions [20] that are more characterized by poor living conditions [27], a known TB risk factor [18], [19].

### 4.1 Limitations

Our study has several limitations that may have influenced findings. Underreporting of TB cases to the NTP and overdiagnosis of TB in the pre-Xpert MTB/RIF period—during which patients with nontuberculous mycobacterium infection may have been misclassified as having TB [30]—could have affected the total number of TB patients.

Also, although over three quarters of patients provided an address in the capital, Paramaribo [25], the city is home to only 45% of Suriname’s population [3]. This discrepancy could be the result of patients being referred or temporarily migrating from rural or interior areas of Suriname to Paramaribo due to lack of local diagnostic and treatment capacity. Underreporting is especially relevant regarding Indigenous people. Despite possessing the highest TB rate found by this study, Indigenous individuals are also more likely to live far removed from the health system. The TB incidence for this group may thus be an underestimate.

Large differences in material poverty exist between ethnic groups in Suriname, with high levels of material poverty observed among Indigenous and Maroon patients [16]. Poverty is an important determinant of TB [19], thus underscoring the importance of patient information pertaining to SES. However, SES data was unavailable in this study.

Data on several factors (e.g., DM status and alcohol consumption) was incomplete and was omitted from analysis. Gaining a better understanding of these factors is important. For example, globally, differences exist between Indigenous and non-Indigenous populations regarding TB risk factors such as DM [31], and elevated DM rates among Indigenous patients in Suriname [13] may help explain their increased vulnerability to TB.

### 4.2 Conclusions

In sum, our retrospective results indicate that Indigenous and Creole individuals had the highest TB incidence in Suriname between 2011 and 2015. HIV, which has an adult (15–49 year) prevalence of 0.9% [12], is an important TB risk factor that does not affect all ethnicities equally. However, data regarding other risk factors (e.g., SES) was limited. We recommend that for better control of TB in Suriname, special attention should be given to the vulnerabilities of different ethnic groups (i.e., frequent monitoring of Indigenous villages in remote areas of Suriname) and to improve access and adherence to antiretroviral therapy for of HIV positive patients. We also recommend that the NTP refine data collection on DM, substance abuse, smoking, and alcohol consumption to make the national TB registry much more complete.

## CRediT authorship contribution statement

**F.A. Gopie:** Conceptualization, Data curation, Investigation, Writing - original draft. **A. Hassankhan:** Writing - review & editing. **S. Ottevanger:** Data curation, Formal analysis, Writing - review & editing. **I. Krishnadath:** Formal analysis, Writing - review & editing. **W. de Lange:** Writing - review & editing. **C.W.R. Zijlmans:** Conceptualization, Supervision, Writing - review & editing. **S. Vreden:** Conceptualization, Supervision, Writing - review & editing.
